# Promoting evidence informed policymaking for maternal and child health in Nigeria: lessons from a knowledge translation workshop

**DOI:** 10.15171/hpp.2018.08

**Published:** 2018-01-07

**Authors:** Chigozie Jesse Uneke, Issiaka Sombie, Henry Chukwuemeka Uro-Chukwu, Yagana Gidado Mohammed, Ermel Johnson

**Affiliations:** ^1^African Institute for Health Policy & Health Systems, Ebonyi State University, PMB 053 Abakaliki, Nigeria; ^2^Organisation Ouest Africaine de la Santé, 175, avenue Ouezzin Coulibaly, 01 BP 153 Bobo-Dioulasso 01, Burkina Faso; ^3^Federation of Muslim Women Association of Nigeria, Bauchi, Nigeria

**Keywords:** Knowledge translation, Policymakers, Researchers, Workshop, Capacity enhancement

## Abstract

**Background:** Knowledge translation (KT) is a process that ensures that research evidence gets translated into policy and practice. In Nigeria, reports indicate that research evidence rarely gets into policymaking process. A major factor responsible for this is lack of KT capacity enhancement mechanisms. The objective of this study was to improve KT competence of an implementation research team (IRT), policymakers and stakeholders in maternal and child health to enhance evidence-informed policymaking.

** Methods:** This study employed a “before and after” design, modified as an intervention study. The study was conducted in Bauchi, north-eastern Nigeria. A three-day KT training workshop was organized and 15 modules were covered including integrated and end-of-grant KT; KT models, measures, tools and strategies; priority setting; managing political interference; advocacy and consensus building/negotiations; inter-sectoral collaboration; policy analysis, contextualization and legislation. A 4-point Likert scale pre-/post-workshop questionnaires were administerd to evaluate the impact of the training, it was designed in terms of extent of adequacy; with “grossly inadequate” representing 1 point, and “very adequate” representing 4 points.

**Results:** A total of 45 participants attended the workshop. There was a noteworthy improvement in the participants’ understanding of KT processes and strategies. The range of the preworkshop mean of participants knowledge of modules taught was from 2.04-2.94, the range for the postworkshop mean was from 3.10–3.70 on the 4-point Likert scale. The range of percentage increase in mean for participants’ knowledge at the end of the workshop was from 13.3%–55.2%.

**Conclusion:** The outcome of this study suggests that using a KT capacity building programme e.g., workshop, health researchers, policymakers and other stakeholders can acquire capacity and skill that will facilitate evidence-to-policy link.

## Introduction

Canada Institutes of Health Research (CIHR) defines knowledge translation (KT) as *“a dynamic and iterative process that includes synthesis, dissemination, exchange and ethically sound application of knowledge to improve the health of people, provide more effective health services and products and strengthen the health care system”*.^1^ KT has been described as a complex multidimensional phenomenon, consequently, a call has been made on the need for better comprehension of its processes including the mechanisms, methods, measurements, and factors influencing it from individual and contextual perspectives.^2^ KT is the meeting ground between research and practice-the two fundamentally different processes that KT has knit together via relationships that can be described as communicative.^3^ KT process is by no means simple because of the involvement of a wide range of interactions between those who produce knowledge (research evidence) and those in position to use knowledge for policymaking. These interactions may vary in the nature and complexity depending on level and extent of engagement of both parties and particularly depending on the needs of the knowledge user in question.^1,4^ Available reports have indicated that successful engagement between policymakers and researchers is anchored on collaborations, networking and some forms of informal relationships between them.^3,5^

In Nigeria, effective policy informed by research evidence is of utmost priority in maternal, newborn and child health (MNCH). Maternal and child health outcome in Nigeria is reportedly poor. Available reports indicate that each year in Nigeria the recorded deaths of newborns, infants and children are above 1 million, the report also showed that in each year up to 50 000 maternal deaths are recorded in Nigeria every year and this is largely attributed to weak health systems.^6,7^ In many low and middle income countries (LMICs) including Nigeria, it is increasingly recognized that research evidence is vital to the formulation of effective policies that can strengthen the health systems.^8,9^ Findings from some previous studies have shown that effective health policy development process can be facilitated by the use of research evidence which can inform the direction and content of a policy decision.^10-13^ There are many potential challenges related to research use in MNCH policymaking and these challenges also affect other aspects of evidence-informed health policymaking process.

Nevertheless, the wide spread failure in most LMICs to uptake many of the high quality research especially in MNCH can be attributed to the lack of capacity of research teams to undertake KT.^14^ For this reason, getting research evidence into policy remains a daunting task.^15,16^ If research teams lack the capacity for KT, they will find it extremely difficult to raise MNCH knowledge users’ awareness of research findings in order to facilitate the use of those findings to improve maternal and child health outcomes.

This is imperative to MNCH research especially in LMICs, because it has been established the implementation of health interventions designed to impact positively on health outcomes is not necessarily dependent on knowledge that is newly created.^1^ A research team with robust KT competence will be able to inspire MNCH stakeholders to use relevant research evidence in the policymaking process and this is usually achieved through regular contact and extensive dialogue between the researchers and policymakers.^14^ In connecting the purity of science with the pragmatism of policy, it is imperative to address the mutual mistrust often existing between policymakers and researchers and institute platforms that will encourage rapport and informal relationships, these can facilitate the evidence to policy link.^3,17^ KT skill is therefore of paramount importance to an MNCH research team as it will enable the team to carefully consider the expectations and policy information needs of policymakers, this can help improve quality of research evidence and transformation of evidence into policy and practice.^18,19^

Available reports in Nigeria, indicate that research evidence rarely gets into the policymaking process.^8,18^ One of the main contributory factors is the lack of KT capacity enhancement mechanisms. There are clear differences between the researchers and the policymakers and some of them are associated with lack of trust and mutual respect for each party, the differences in career requirement and path, the disposition towards information etc.^19^ The persistence of these differences is affecting the process of getting research evidence into policy. It is further compounded by the lack of platforms or forum that will bring researchers and policymakers together to consider issues around research-to-policy-to-practice link.^20,21^

In this report, we present the outcome of a training workshop designed to enhance the capacity of an MNCH implementation research team (IRT) to effectively undertake KT and to promote evidence informed policymaking and improve maternal and child health outcomes in Nigeria.

## Materials and Methods

### Study design

This study employed a “before and after” design, modified as an intervention study as described in previously.^22^ The study was designed as a three-day KT training workshop organized into fifteen modules including: **i**ntegrated and end-of-grant KT; KT models, measures, tools and strategies; priority setting; managing political interference; advocacy and consensus building/negotiations; inter-sectoral collaboration; policy analysis, contextualization and legislation. A 4-point Likert scale pre-/post-workshop questionnaires were administered to evaluate the impact of the training, it was designed in terms of extent of adequacy; with “grossly inadequate” representing 1 point, and “very adequate” representing 4 points.

### Workshop attendees profile

The workshop took place in November 2016 in Bauchi the capital of Bauchi State located in the northeastern Nigeria. The improvement of the health of women of child bearing age and children is a topmost agenda in the Nigerian State of Bauchi, because maternal and child health outcomes in the State are among the poorest in Nigeria. According to available report, the maternal mortality ratio (MMR) in Bauchi is 1540 per 100 000 live births, while the infant mortality rate (IMR) is 78 per 1000 live births.^23^ A total of 45 individuals took part in the workshop. Table 1 presents the profile of the attendees. These included members of Bauchi State MNCH IRT, members of the project management team, project steering committee, State primary health care development agency (SPHCDA) staff, Ministry of Health (MOH) staff, health board members of Federation of Muslim women association of Nigeria (FOMWAN), officials of non-governmental organizations (NGOs) and Local government authority (LGA) health personnel. The IRT is undertaking an implementation research in which edutainment strategy is employed as a tool for the improvement of access to quality maternal and child health care in rural areas of Bauchi State.

### Workshop content and pattern

The workshop was developed as a 3-day training event. The workshop package consisted of 15 modules, designed to enhance the KT competence of the IRT and other stakeholders closely associated with the research undertaken by the IRT. Five modules were taught each day (Tables 1-3). The workshop consisted of lecture sessions and group work sessions. Power-point presentation was used for the teachings and handouts were provided for participants on each module treated. Focus group discussions, dialogues, question/answer sessions and group work were held.

### Pre-post workshop questionnaire 

A pre-workshop questionnaire was administered before the commencement of the training each day. The questions contained in the pre-workshop questionnaire were designed to evaluate the extent of participants’ initial knowledge/understanding of the modules. At the end of each day’s training a similar questionnaire was given to the participants to evaluate the post workshop understanding of the modules taught.

The data collection questionnaires used for the pre/post workshop assessment were designed as structured questionnaires. They scale, number of items, title of domains, and scoring mode were modified from the evaluation tool by Johnson and Lavis^24^ and the Canadian Health Services Research Foundation (CHSRF) (http://www.chsrf.ca/other_documents/working_e.php). Our choice of the self-assessment tool of CHSRF was because several previous studies showed that the tool is very useful and reliable in the evaluation of health stakeholders’ capacity to use evidence from research for policymaking and practice.^25-27^ The questionnaires were pre-tested and validated in our previous evidence-to-policy training workshops for policymakers and other stakeholders in the health sector and found to be relaible.^20,28-30^ The final version of the validated questionnaire was used for the present study.

### Analysis of questionnaire

We employed the Johnson and Lavis^24^ method in the analysis of the completed questionnaires, which stipulates the use of mean rating (MNR). Details of the methods is provided in our previous studies.^20,28-30^ The percentage differences in the MNR of the pre-workshop and post-workshop were calculated and used as indicator of the extent of improvement in the participants knowledge after the training.

## Results

A total of 45 individuals participated in the workshop. Of these 28.9% were females. Up to 19 participants were from the ministry of health and its associated agencises including, State primary health care development agency 4 (8.9%), ministry of health 6 (13.3%) and the Local government area health unit 9 (20%) (Table 1).

The outcome of the analysis of the pre-workshop and post-worshop questionnaire showed remarkable increase in understanding of the modules taught, as demonstrated by noteworthy improvement the percentage mean ratings. The range of the mean of pre-workshop understanding of the modules was 2.04-2.94, but the range of the mean of the postworkshop understanding was considerably higher at 3.10–3.70 on the Likert scale of 4 points. The range of the mean percentage increase in participants knowledge/understanding of the modules taught was from 13.3%–55.2%.

In Table 2, the mean rating percentage improvement for the modules were: Introduction to health policy & health systems (24.0%-37.5%); Introduction to knowledge translation (integrated KT & End-of-Grant KT) (35.0%-55.2%); Research priority setting (32.9%-45.5%); Leadership capacity development & managing political interference (13.3%-31.7%); Getting research into policy and practice (19.9%-45.6%).

In Table 3, the mean rating percentage improvement for the modules were: KT models and measures (26.7%-32.2%); Research evidence in health policy making and health policy implementation (17.8%-28.5%); Health policy advocacy, demand creation, consensus building and negotiations (19.1%-24.5%); KT tools and strategies for stakeholders and end users engagement (24.9%-34.9%); Policy formulation and implementation process (25.1%-38.4%)

In Table 4, the mean rating percentage improvement for the modules were: Policy review, analysis and contextualization (24.8%-34.5%); Inter-sectoral collaboration in policymaking & implementation (19.6%-37.3%); Knowledge dissemination, exchange & management (20.1%-25.6%); Health policy monitoring, evaluation and performance assessment (18.3%-22.1%); Introduction to policy legislation (28.9%-31.3%).

## Discussion

The findings of the present study suggest that a KT training workshop has the potential to serve as a vital platform to improve the understanding and skill of researchers and policymakers regarding evidence to policy process. The workshop brought together both researchers and policy makers and afforded them the opportunity to interact and considers issues around MNCH research-to-policy link. This type of capacity enhancement forum has been shown to be very critical to the process of bridging the gap between research and policy/practice.^8,9^ According to Haines and colleagues,^14^ in order to enhance research finding uptake into policy and practice, it is imperative to strengthen mechanisms and platforms that can promote the systematic interactions between researchers and policymakers. Furthermore, Choi et al^31^ argued that researchers and policymakers will appreciate their differences better (in terms of their career paths, goals, disposition to evidence etc), if greater number of collaborative opportunities are created for them to interact, and will improve evidence to-policy link.

In this study we introduced 15 modules into the workshop curriculum. The topics were carefully designed to enable participants to have a better understanding of the non-linear but rather complex process of getting evidence into policy and practice. It is well established that uptake of evidence into mainstream policy/decision making is a very complex nonliner process frequently involving political and other forms of interferences and influences.^32^ Furthermore, Green and Bennett^33^ argued that at all levels, political, social and economic factors strongly influence who makes policies, how and where the policies are made. Consequently, the understanding of KT process cannot be complete without taking into consideration intertwined sets of influences such as leadership and governance; policy contextualization; priority setting; advocacy/consensus building, etc.

We incorporated a module on inter-sectoral collaboration because KT can never become impactful without intersectoral collaboration. The scarcity of resources is a major factor that necessitates intersectoral collaboration in order to avoid unnecessary waste of resources in policymaking and implementation. Intersectoral collaboration is critical to KT because certain elements of collaborative efforts such as having similar objectives/goals, resource interdependence, leadership that is facilitative are among the key KT success factors.^1,34^

Another crucial component of the KT workshop was the module on stakeholders and end users engagement. We included this module because of the need for the IRT to be equipped with skill for stakeholders engagement which is critical to KT. According to CIHR,^1^ one of the most important elements in KT is that it promotes active engagement of knowledge users by researchers and provides them the platform to collaborate as equal partners. This arrangement has in many instances resulted in the execution of research that are more relevant to the needs of policymakers and greatly facilitated their uptake in policymaking.^1^

Thus, to achieve KT, it is imperative for researchers to understand that every stage of research process presents a very valuable opportunity for them to partner with the policymakers. The CIHR noted that the research stages where stakeholders’ engagement is imperative include the development of research questions, identification of methods, development of data collection instruments and tools, data collection and analysis, interpretation of findings and dissemination.^1^ These were emphasized in this study during the workshop.

Emphasis were also placed on policy legislation and managing political interference during the training because of the strategic role both issues play in the KT process. According to Clarke,^35^ policy legislation is very critical in the translation of policy objectives into action, and this is because policy legislation can make provision for the use of sanctions and incentives to facilitate policy implementation and compliance. The importance of understanding the legislative process in KT process cannot be overstated because it is imperative for policymakers and researchers participating in policymaking to have adequate knowledge of legislative requirements that guide policymaking and policy implementation.^35^

The module on managing political interference was incorporated into the KT training workshop because of the important role political context plays in the policymaking process. A previous report, described the policymaking process as highly political because the competing and conflicting interests, values and ideologies of the key actors involved in the process.^33^ Prewitt^36^ in his book ‘*Winning the Policy War’* argued for a metaphoric change of terminology from evidence-based policy to evidence-influenced politics because of the critical role played by politics in the policymaking process. The Overseas Development Institute (ODI), had in a previous report described the process of getting research evidence into policymaking and practice as purely political from start to finish.^37^ Understanding the role politics plays in the policymaking process is therefore critical to the success of KT.

The post workshop assessment showed percentage increase ranging from 13.3% to as high as 55.2% in the understanding of all the modules. This clearly suggests that the workshop had a remarkable impact in terms of improving the understanding of the participants regarding KT. A number of similar previous training workshops that brought together researchers and policymakers as participants resulted in significant improvement in the understanding of the participants regarding evidence to policy process.^38-40^ The strategies we employed in the training workshop including completion of pre/post-workshop questionnaires and group works have been shown to facilitate knowledge enhancement.^41^

Among all the modules taught, result showed the highest percentage improvement in participants’ understanding of the integrated KT (iKT) and end-of-grant KT (eKT) module (35.0%-55.2%). This clearly suggests that prior to this workshop, the understanding of the participants regarding the meaning and core principles of KT, models of KT and iKT/eKT was inadequate. This outcome is encouraging as it suggests that the IRT of Bauchi State Nigeria will be more likely to adapt the KT principles and practices for better implementation of their research project and engage the policymakers through out the various stages of their research to facilitate uptake of the evidence in policy development. According to Tchameni Ngamo and colleagues,^42^ adequate understanding of KT will enable a research team to give attention to policymakers needs throughout the research process thereby enhancing the impact of the policy.

## Study limitations

In this study we used the self-assessment method to assess the impact of the workshop. Although this technique has some merits, its weakness and limitation lie in the fact that it is difficult for an individual to provide an accurate and unbiased self-assessment of one’s deficiencies in knowledge and skill.^43^ Self-assessments have also been described as highly subjective, not very reliable and cannot easily be validated.^44^ Secondly, the evaluation period was rather too short (only 3 days) to ascertain the true impact of the training workshop. A follow-up of the IRT and monitoring of their implementation research activity over a period of time is necessary to ascertain their employment of the KT strategies learnt from the training. These limitations notwithstanding, the study has shown that a KT training workshop has the potential of improving the understanding and knowledge of both researchers and policymakers regarding evidence-formed policymaking process. We recommend similar KT training workshop for IRTs in low income settings.

## Ethical approval

Ebonyi State University Research Ethics Committee (UREC) provided approval and ethical clearance for the study. The UREC guideline which stipulates voluntary participation, obtaining of informed consent, maintenance of anonymity of participants and the confidential treatment of all participant related information were strictly adhered to. The study informed consent form was signed by participants after completing it.

## Competing interests

No competing interest is declared by authors.

## Authors’ contributions

The design, development and execution of this study were undertaken by all authors. The manuscript was drafted by CJU, various inputs were made by all other authors. The final manuscript was approved by all authors.

## Acknowledgments

The International Development Research Centre (IDRC) Canada provided funding support to this study through the West African Health Organization (WAHO) (Reference: IDRC 107892_001). We thank the researchers, policymakers and other Nigeria MNCH stakeholders who took part in this investigation.

**Table 1:** Profile of participants at the knowledge transition workshop Bauchi Nigeria (N = 45)

**Parameter assessed**	**No. (%)**
Gender	
Male	32 (71.1)
Female	13 (28.9)
Participants organization	
Bauchi IRT	8 (17.8)
Project management/steering committee	5 (11.1)
SPHCDA Staff	4 (8.9)
NGO/CSO	3 (6.7)
Ministry of Health Staff	6 (13.3)
FOMWAN Health Board	5 (11.1)
LGA Health Staff	9 (20.0)

Abbreviations: IRT, implementation research team; SPHCDA, state primary health care development agency; NGO/CSO, non-governmental organization/civil society organization; FOMWAN, Federation of Muslim Women Association of Nigeria; LGA, local government area.

**Table 2:** Outcome of the pre-workshop and post-workshop questionnaire analyais for day 1 of the KT training workshop in Bauchi Nigeria

**Parameters assessed**	**Pre-workshop mean**	**Post-workshop mean**	**% Mean increase**
Introduction to health policy & health systems			
Knowledge of the meaning of policy and policy cycle	2.54	3.39	33.5
Understanding of the critical policy issues and the focus/forms of policy analysis	2.50	3.10	24.0
Understanding of building blocks of the health systems	2.56	3.52	37.5
Introduction to knowledge translation (integrated KT & End-of-Grant KT)			
Knowledge of the meaning and core principles of knowledge translation	2.34	3.16	35.0
Understanding of the four models of knowledge translation	2.21	3.27	48.0
Understanding of iKT and eKT	2.10	3.26	55.2
Research priority setting			
Knowledge of the principles and essential elements of policy research priority setting process	2.21	3.26	45.5
Understanding of the value of public engagement in policy research priority setting process	2.55	3.39	32.9
Understanding of the criteria for priority setting and the process of convening a policy research priority setting exercise	2.25	3.17	40.9
Leadership capacity Development & managing political interference			
Knowledge of the contextual issues about policymaking sector leadership	2.43	3.20	31.7
Understanding of policymakers’ leadership capacity development process	2.59	3.28	26.6
Understanding of leadership characteristics for successful policymakers	2.54	3.32	30.7
Knowledge about managing political interference in policymaking and implementation	2.71	3.07	13.3
Knowledge about managing political interference in policymaking and implementation	2.38	3.13	31.5
Getting research into policy and practice			
Understanding of critical policy issues and the focus/forms of policy analysis	2.11	2.96	40.3
Understanding of the concept of policy process and policy assistance	2.41	2.89	19.9
Understanding of research to policy inter-face and systems thinking	2.04	2.97	45.6

Abbreviations: iKT, integrated knowledge translation; EKT, end-of-grant knowledge translation.

**Table 3:** Outcome of the pre-workshop and post workshop questionnaire analyais for DAY 2 of the KT training workshop in Bauchi Nigeria

**Parameters assessed**	**Pre-workshop mean**	**Post-workshop mean**	**% Mean increase**
Knowledge translation models and measures			
Knowledge of the characteristics of knowledge translation	2.66	3.41	28.2
Understanding of the frameworks applicable to knowledge translation	2.58	3.41	32.2
Understanding of knowledge management and the strategies	2.81	3.56	26.7
Research evidence in health policy making and health policy implementation			
Knowledge of the quality and relevance of the evidence	2.63	3.38	28.5
Understanding of the role of research evidence in informing health policy decisions	2.73	3.39	24.2
Understanding of use of evidence in health policy implementation	2.92	3.44	17.8
Health policy advocacy, demand creation, consensus building and negotiations			
Knowledge of advocacy strategies	2.82	3.36	19.1
Understanding of constituency-building and resource mobilization	2.76	3.35	21.8
Understanding of the principles of demand creation	2.69	3.35	24.5
Knowledge translation tools and strategies for stakeholders and end users engagement			
Understanding of the tools for knowledge translation and exchange	2.61	3.52	34.9
Knowledge of the preparation and key ingredients of effective policy brief	2.64	3.42	29.5
Understanding of the need and characteristics of policy dialogue	2.85	3.56	24.9
Policy Formulation and Implementation Process			
Knowledge of the meaning and elements of policy	2.63	3.29	25.1
Understanding of policy cycle	2.61	3.47	33.0
Understanding of the concept of policy process and policy assistance	2.45	3.39	38.4

**Table 4:** Outcome of the pre-workshop and post workshop questionnaire analyais for DAY 3 of the KT training workshop in Bauchi Nigeria

**Parameters assessed**	**Pre-workshop mean**	**Post-workshop mean**	**% Mean increase**
Policy review, analysis and contextualization			
Knowledge of the policy review process	2.86	3.57	24.8
Understanding of the success factors for multi-stakeholder policy review methods	2.81	3.57	27.0
Understanding of review tasks to guide the multi-stakeholder review	2.75	3.70	34.5
Inter-sectoral collaboration in policymaking & implementation			
Knowledge of the meaning of inter-sectoral collaboration in policymaking & implementation	2.70	3.60	37.3
Understanding of what makes collaboration work	2.91	3.48	19.6
Understanding of the roadblocks to effective collaboration	2.73	3.57	30.8
Knowledge dissemination, exchange & management			
Knowledge of fundamentals and approaches of knowledge dissemination	2.92	3.57	22.3
Understanding of knowledge exchange and what makes the integrated KT process work effectively	2.81	3.53	25.6
Understanding of the effective ways of disseminating policy information	2.94	3.53	20.1
Health policy monitoring, evaluation and performance assessment			
Understanding of value of policy monitoring and evaluation	2.81	3.43	22.1
Understanding of the concept of policy process and policy assistance	2.81	3.38	20.3
Knowledge about steps to building a performance based monitoring and evaluation system	2.73	3.23	18.3
Introduction to policy legislation			
Knowledge of the meaning of a bill for legislation	2.65	3.48	31.3
Understanding of the mechanism of the development of a bill	2.70	3.48	28.9
Understanding of the bill and legislative process at the House of Assembly	2.69	3.48	29.4
General Questions on the training workshop outcome			
Facilitators’ mastery & ability to deliver the lessons in an understandable manner		3.79	
Scope/coverage of the training workshop in relation to health policy and knowledge translation		3.55	
Duration of the programme sufficient to address major individual knowledge & capacity constraints in evidence-informed health policymaking		2.93	
Workshop assessment			
Overall assessment of the training workshop	41%-60%	61%-80%	81%-100%
Participants score	3.7	25.9	70.4

## References

[1] Canada Institutes of Health Research (CIHR). Guide to Knowledge Translation Planning at CIHR: Integrated and End-of-Grant Approaches. Ottawa, Canada: CIHR; 2012.

[2] Sudsawad P. Knowledge translation: Introduction to models, strategies, and measures. Austin, TX: Southwest Educational Development Laboratory, National Center for the Dissemination of Disability Research; 2007.

[3] Bennett G, Jessani N. The Knowledge Translation Toolkit-Bridging the Know-Do Gap: A Resource for Researchers. London, UK: SAGE Publications Ltd; 2011.

[4] Ongolo-Zogo P, Lavis JN, Tomson G, Sewankambo NK (2015). Climate for evidence informed health system policymaking in Cameroon and Uganda before and after the introduction of knowledge translation platforms: a structured review of governmental policy documents. Health Res Policy Syst.

[5] Makkar SR, Williamson A, Turner T, Redman S, Louviere J (2015). Using conjoint analysis to develop a system of scoring policymakers’ use of research in policy and program development. Health Res Policy Syst.

[6] Federal Ministry of Health. Integrated Maternal, Newborn and Child Health Strategy. Abuja: Federal Ministry of Health; 2007.

[7] National Population Commission Nigeria and ICF International. Nigeria Demographic and Health Survey 2013. Abuja, Nigeria: NPC and ICF International; 2014.

[8] Uneke CJ, Ezeoha AE, Ndukwe CD, Oyibo PG, Onwe F (2012). Promotion of evidence-informed health policymaking in Nigeria: bridging the gap between researchers and policymakers. Glob Public Health.

[9] Uneke CJ, Ezeoha AE, Ndukwe CD, Oyibo PG, Onwe F (2010). Development of health policy and systems research in Nigeria: lessons for developing countries’ evidence-based health policy making process and practice. Healthc Policy.

[10] Innvaer S, Vist G, Trommald M, Oxman A (2002). Health policy-makers’ perceptions of their use of evidence: a systematic review. J Health Serv Res Policy.

[11] Hanney SR, Gonzalez-Block MA, Buxton MJ, Kogan M (2003). The utilisation of health research in policy-making: concepts, examples and methods of assessment. Health Res Policy Syst.

[12] Dobrow MJ, Goel V, Upshur RE (2004). Evidence-based health policy: context and utilisation. Soc Sci Med.

[13] Campbell DM, Redman S, Jorm L, Cooke M, Zwi AB, Rychetnik L (2009). Increasing the use of evidence in health policy: practice and views of policy makers and researchers. Aust New Zealand Health Policy.

[14] Haines A, Kuruvilla S, Borchert M (2004). Bridging the implementation gap between knowledge and action for health. Bull World Health Organ.

[15] Young J (2005). Research, policy and practice: why developing countries are different. J Int Dev.

[16] Jonsson K, Tomson G, Jonsson C, Kounnavong S, Wahlstrom R (2007). Health systems research in Lao PDR: capacity development for getting research into policy and practice. Health Res Policy Syst.

[17] Makkar SR, Williamson A, Turner T, Redman S, Louviere J (2015). Using conjoint analysis to develop a system to score research engagement actions by health decision makers. Health Res Policy Syst.

[18] Canadian Institute of Health Research ( CIHR). Developing a CIHR framework to measure the impact of health research (cihr synthesis report). In: CIHR. More about knowledge translation at CIHR. Ottawa: CIHR; 2005.

[19] Landry R, Lyons R, Amara N, Warner G, Ziam S, Halilem N, et al. Knowledge Translation Planning Tools for Stroke Researchers. Available from: http://www.ahprc.dal.ca/pdf/kt/2006_KTP lanningTool.pdf. Accessed August 2014.

[20] Uneke CJ, Ezeoha AE, Ndukwe CD, Oyibo PG, Onwe F, Igbinedion EB (2011). Individual and organisational capacity for evidence use in policy making in Nigeria: an exploratory study of the perceptions of Nigeria health policy makers. Evidence Policy: A Journal of Research, Debate and Practice.

[21] Uneke CJ, Ndukwe CD, Ezeoha AA, Uro-Chukwu HC, Ezeonu CT (2015). Implementation of a health policy advisory committee as a knowledge translation platform: the Nigeria experience. Int J Health Policy Manag.

[22] Purdon S, Lessof C, Woodfield K, Bryson C. ‘Research Methods for Policy Evaluation’, DWP Social Research Division Working Paper 2. London: Social Research Branch; 2001.

[23] Abegunde D, Orobaton N, Sadauki H, Bassi A, Kabo IA, Abdulkarim M (2015). Countdown to 2015: tracking maternal and child health intervention targets using lot quality assurance sampling in Bauchi State Nigeria. PLoS One.

[24] Johnson NA, Lavis JN. Procedures Manual for the “Evaluating Knowledge-Translation Platforms in Low- and Middle-Income Countries” Study. Hamilton, Canada: McMaster University Program in Pol-icy Decision-Making; 2010.

[25] Canadian Health Services Research Foundation (CHSRF). Validating the Foundation’s self-assessment tool: A summary. Ottawa, ON: CHSRF; 2008.

[26] Thornhill J, Judd M, Clements D (2009). CHSRF knowledge transfer: (re)introducing the self-assessment tool that is helping decision-makers assess their organization’s capacity to use research. Healthc Q.

[27] Shroff Z, Aulakh B, Gilson L, Agyepong IA, El-Jardali F, Ghaffar A (2015). Incorporating research evidence into decision-making processes: researcher and decision-maker perceptions from five low- and middle-income countries. Health Res Policy Syst.

[28] Uneke CJ, Sombie I, Keita N, Lokossou V, Johnson E, Ongolo-Zogo P (2017). Improving maternal and child health policymaking processes in Nigeria: an assessment of policymakers’ needs, barriers and facilitators of evidence-informed policymaking. Health Res Policy Syst.

[29] Uneke CJ, Ezeoha AE, Uro-Chukwu H, Ezeonu CT, Ogbu O, Onwe F (2015). Enhancing health policymakers’ information literacy knowledge and skill for policymaking on control of infectious diseases of poverty in Nigeria. Online J Public Health Inform.

[30] Uneke CJ, Sombie I, Keita N, Lokossou V, Johnson E, Ongolo-Zogo P (2016). An assessment of national maternal and child health policy-makers’ knowledge and capacity for evidence- informed policy-making in Nigeria. Int J Health Policy Manag.

[31] Choi BC, Gupta A, Ward B (2009). Good thinking: six ways to bridge the gap between scientists and policy makers. J Epidemiol Community Health.

[32] Jones N, Walsh C. Policy briefs as a communication tool for development research: Background note. London: Overseas Development Institute; 2008.

[33] Green A, Bennett S. Sound Choices: Enhancing Capacity for Evidence-Informed Health Policy. Geneva: World Health Organization; 2007.

[34] Miller R. Cooperation, Coordination, and Connection: Evaluating the Effectiveness of Intersectoral Collaboration through the Lens of Stormwater Management [dissertation]. Ypsilanti: Eastern Michigan University, Department of Political Science; 2009.

[35] Clarke D. Chapter 10. Law, regulation and strategizing for health. In: Schmets G, Rajan D, Kadandale S, eds. Strategizing National Health in the 21st Century: A Handbook. Geneva: World Health Organization; 2016.

[36] Prewitt K. Winning the Policy Wars. London, UK: Global Public Policy Network, London School of Economics and Political Sciences; 2006.

[37] Overseas Development Institute (ODI). Bridging research and policy in international development: an analytical and practical framework. Research and Policy in Development Programme Briefing Paper No. 1. London: Overseas Development Institute; 2004.

[38] Sabet Z. Policymakers address researchers at the GDNet-AERC Policy Brief Training Workshop. Research to Action; 2011. Available from: http://www.researchtoaction.org/2011/12/policymakers-address-researchers-at-the-gdnet-aerc-policy-brief-training-workshop/. Accessed Feb 2, 2017.

[39] Oronje RN. Using training as one approach for building the capacity of health policymakers in evidence-informed policymaking: Recent experiences and reflections from Kenya and Malawi. African Institute for Development Policy; 2015. https://www.afidep.org/?p=2164. Accessed Feb 2, 2017.

[40] African Institute for Development Policy. SECURE Health Conducts Evidence-Informed Policymaking Training for Technocrats in the Ministries of Health and Parliaments in Kenya and Malawi. African Institute for Development Policy; 2015. Available from: http://dspace.africaportal.org/jspui/bitstream/123456789/35682/1/secure%20health%20news%202.pdf?1. Accessed Feb 2, 2017.

[41] Poulos RG, Zwi AB, Lord SR (2007). Towards enhancing national capacity for evidence informed policy and practice in falls management: a role for a “Translation Task Group”?. Aust New Zealand Health.

[42] Tchameni Ngamo S, Souffez K, Lord C, Dagenais C (2016). Do knowledge translation (KT) plans help to structure KT practices?. Health Res Policy Syst.

[43] Deans F, Ademokun A. Investigating capacity to use evidence. Available from: http://www.inasp.info/uploads/filer_public/2013/07/04/investigating_capacity_to_use_evidence.pdf. Accessed Feb 2, 2017.

[44] Haahr JH, Shapiro H, Sørensen S. Defining a Strategy for the Direct Assessment of Skills. Danish Technological Institute; 2004. Available from: http://www.pedz.uni-mannheim.de/daten/edz-b/gdbk/04/ defining_strategy_final.pdf. Accessed Feb 2, 2017.

